# Conversations upon the Physical and Mental Hygiene of Girlhood

**Published:** 1881-07-20

**Authors:** T. S. Powell

**Affiliations:** Atlanta; Professor of Obstetrics and Diseases of Women, and Lecturer on Medical Ethics in the Southern Medical College


### Southern Medical Record:

EDITORS:

T. S. POWELL, M.D. W. T. GOLDSMITH, M.D. R. C. WORD, M.D.

*R. C. WORD, M.D., Managing Editor.*

☞All Communications and Letters on Business connected with the RECORD must be addressed to the Managing Editor.

VOL. XI. ATLANTA, GA., JULY 20, 1881. NO. 7.

### ORIGINAL AND SELECTED ARTICLES.

### CONVERSATIONS UPON THE PHYSICAL AND MENTAL HYGIENE OF GIRLHOOD.

BY T. S. POWELL, M. D.,

Professor of Obstetrics and Diseases of Women, and Lecturer on Medical Ethics in the Southern Medical College.

### CONVERSATION VI.

Doctor—"Good morning, madam; how time flies; I believe it has been nearly two weeks since my last visit."

Mother—"Yes, and I have been expecting you for several days, Doctor, and now that you have come, I am glad to see you."

Doctor—"Thank you madam, though doctors are told that every day, yet it makes them feel as a lady does when she hears she is handsome, peculiarly pleasant. How is Miss Mary? I presume you have heard from her since I saw you?"

Mother—"Yes, I have received one or two notes from her since then, and she reports a continual improvement in health. She requested me to tell you that she is following your instructions, walks several miles every day, romps and plays, and with good, well-cooked food, pure spring water, and plenty of sound sleep at night, she is gaining flesh, and really growing fat, compared to her weight when I stopped her from school."

Doctor—"I know how happy such news makes you feel, my dear madam, and I rejoice with you in your daughter's improvement. It will probably be several months before her catamenia is well established and regular, but I feel assured that in six or eight months she will be restored to perfect health."

Mother—"I indeed hope so; but, doctor, I still cannot think of the distressing and dangerous condition she was in without reproaching myself for my shameful ignorance as a mother, and feeling indignant with the errors in our public schools that so damaged my daughter's health."

Doctor—"While that may be true, madam, our public schools do a great deal of good, and it is our duty to work with their friends to correct all these errors; and as I said at my last visit, I am sure that this reform will yet take place. The good time is coming when the science of disease and preservation of health will be so well understood by teachers and parents, the people will know how to prolong life to extreme old age, and then die in possession of all their faculties, and without pain, as if really falling to sleep. This I believe is the design of the Creator, and as there is no such thing as science without interpretation by the light of Christianity, the people should all be taught that the culture and preservation of a sound mind in a sound body is not only a family, a social and public duty, but it is one of moral and religious obligation. No sensible person can think for a moment, and then deny that the cleaner and purer the body and mind are kept here in life, the better fitted we will be to inhabit that world where there is no disease or death."

Mother—"Yes, I should think so, and if we only knew how to keep a sound mind in a sound body, I do not think we ought to look upon it as so much of an obligation as a great privilege, and be very grateful for the knowledge, since it will add so much to our success, usefulness and happiness even in this life."

Doctor—"Very true, madam; but it is the proper education that will enable the people to look upon these things as high privileges as well as obligations. Men should learn the laws of vitality as well as those which govern mechanical, chemical, or financial forces. The first is indeed the more important, and when they acquire the knowledge they will then understand how to rest both body and mind, how to nourish and keep them in a state of health, vigor and perfection."

Mother—"Yes, doctor, if we all knew and practiced these things, what an improved people we soon would be."

Doctor—This knowledge can be acquired only by being taught the chemical and hygienic laws that will enable the people to know which are the best elements of nutrition in food, and what are the most important factors for promoting growth, physical power, and perfect health according to the various ages and conditions of the individuals."

Mother—"And while this is all so important, I do not suppose one mother, or her cook, out of a hundred know anything about what is nutritious food, and what is not."

Doctor—"No, madam, I suppose not, and yet you see the imperative necessity for such knowledge. For instance, milk is the best food for children up to two or three years of age, because it furnishes the best proportioned combination of nitrogenous and carbonaceous elements which are necessary to develop nerve and muscular power, and produce firm, sound flesh rather than an excess of fat, which is not a healthy condition for a child under three or four years."

Mother—"Why, doctor, I thought the fattest babies were the healthiest."

Doctor—"No, madam, that is another popular error. The weight of the child is not so important as the quality of its flesh. If, as I have said, this is firm and good in due proportion to the child's age, and its nerve and muscular force is well devoloped, it will not be so liable to the many diseases incident to infancy, and they will not be so apt to prove fatal. Chemistry teaches us that pure, healthy milk is the best food for young children; so good corn meal, unbolted wheat meal and butter contain the same important elements to preserve the health and vigor of adults. This diet contains about four times as much carbon and a little more than four times as much nitrogen as is contained in good beef."

Mother—"And for that reason I suppose growing children could really thrive upon this diet without any meat."

Doctor—"Yes, madam, they certainly could, though after a child is three or four years of age, if it is in good health, there is no objection to its eating a little tender, healthy beef, mutton, or game, at the proper time, and properly cooked. A knowledge of all these things, as I have just intimated, will yet be taught in our public schools, and the masses of the people will know how to promote their health, happiness and longevity."

Mother—"I hope that time will really come, and very soon."

Doctor—"It will be done just as soon as these reforms meet with the intelligent co-operation of the people, but we cannot expect it from them until they are educated so as to see and appreciate the great need of such a movement. When the people begin to know that it is more important to understand the laws that govern the growth and vigor of their children and themselves than it is to successfully grow fine grain, cotton, plants and flowers, then they will be ready to heartily co-operate in bringing about this reform."

Mother—"Yes, when they receive such an education, then we can confidently expect corresponding results."

Doctor—"We must continue to wait and hope, and at the same time labor for these results. The light of science will yet dispel all this darkness of ignorance, and the causes that produced your daughter's ill health will be removed to a great extent; but those of a general nature, such as we have already mentioned—impurities of air, bad water, unsound food, improper cooking, suitable clothing, and the proper exercise adapted to the ages of children and their conditions of health—these must all be looked after and regulated by both the mother and the father of the children when at home."

Mother—"Yes, while I know the responsibility of a girl's health rests upon her teachers to a great extent, that of the parents is still greater."

Doctor—"And for that reason, while it is true that often young girls of the fairest promise die early, or spend a life of miserable invalidism, it is important and very dificult to decide whether the health of girls is not as frequently destroyed by the ignorance, and sometimes the wilful ignorance and obstinacy of parents, as by any of the laws or regulations that are required to be observed at school. These errors on the part of parents and school systems affect the health of girls in both mind and body differently. In your daughter's case, the mind was over-taxed by constant mental work, and body impoverished by imperfect nutrition, but I have seen many cases from the same cause where the body developed and continued in a healthy condition, and the mind failed entirely or had its power greatly weakened. I could call to mind several instances of both boys and girls, who in their childhood promised to be, or were unusually bright in intellectual capacity, but now at sixteen or eighteen have lost the mental power and vivacity that distinguished them at ten years of age. In others the mind grows and increases in force, while the body ceases to grow or falls into incurable invalidism, which would have been the fate of your daughter if her case had not been attended to before it was too late."

Mother—"Yes, I now feel satisfied of that fact, and it has indeed taught me a lesson I can never forget."

Doctor—"Our present system of education, aided by the ignorance, the over-indulgence, and cruel as well as fatal ambition of parents to see their children "advance" rapidly, furnish many instances of both these classes of disease. We very often hear that one or more of our first honor girls have died of a fever or some other simple acute disease, because the system was so enfeebled by long and continued study, it had not enough strength and vitality to resist any ordinary disease.

Growing girls are feebler and more sensitive than boys, physically and mentally, and they should be treated as growing girls until they are at least eighteen, otherwise they will certainly have an imperfect womanhood. Though, as I have said, boys are frequently and fatally over-taxed with mental work, yet they have more firmness of fibre than girls, and which gives strength and endurance to the body and brain."

Mother—"When I was growing up I thought no one but aged, feeble persons had what we call nervous diseases, but I now know that young persons, even children, are very frequently victims of these distressing disorders."

Doctor—"Yes madam, many of them. The same causes we have been discussing—mental and physical taxation—by lessening the vital energies and the resisting powers of economy, not only predispose to, and makes it more difficult to cure acute disease, but in my judgment is frequently the cause of many diseases. I have had four cases of Chorea or St. Vitus Dance out of one school, resulting, as I have reason to believe, from over-taxing the nervous system, and from the mental anxiety to which children are subjected in their education."

Mother—"I am not at all surprised to hear it when the children's nerves are kept in a perpetual strain from the time they awake in the morning until they fall into a sleep of exhaustion at night. I know just how they do, Doctor, for I have seen it in my own children. It is hurry when they are dressing in the morning for fear they will be "tardy," and hurry all the time until they get off. If they go to school too late, they get a "demerit," they get one if they go too early, and in anticipation of it they are in a fidget, a distressing, restless state until they work themselves up to excessive nervous excitement, then swallow their breakfast in haste, go to school almost in a run, get there panting and excited, and in a *nice* condition for solid, deliberate and thorough study."

Doctor—"Yes, madam, I have heard other mothers say the same thing in regard to their children."

Mother—"I actually heard the other day of a party of children who came very near being crushed to death by the cars, because they were afraid they would reach school too late and get the inevitable demerit; they would not wait for the train to pass at the crossing; and they escaped death so narrowly, a gentleman near by, shuddering at the danger they had been in, spoke to them about it, when one of the party said, 'I had rather be *killed* than get to school too late!' Is not that shocking?"

Doctor—"It is, madam. It seems the children now in our towns and cities fear the mark of demerit more than the little people used to care for a good old-fashioned switching at school in the times gone by."

Mother—(laughing). "I suppose it was because they did not know then when the switching was coming, and their minds and nerves, therefore, did not suffer the tortures of its anticipation. And when they did get the punishment, it was done with at once, and the nervous system left free to recover its tone."

Doctor—"Yes, madam, I admit there is some philosophy in your supposition."

Mother—"I also think, Doctor, that this custom in schools of giving prizes and medals is injurious to pupils under seventeen or eighteen years of age. It stimulates their ambition to such an extent that their mental anxiety is extreme, and consequently the nervous system is strained to its utmost capacity. So, in reaching brilliant achievements in scholarship, if the mind is not injured, the physical vitality is enfeebled if not exhausted. Do you not think so, Doctor?"

Doctor—"Of course, madam, it is only another instance of excessive mental taxation, and when it is kept up for weeks and months together, no matter what the object is in view, the physical results cannot be beneficial."

Mother—"Doctor, I will tell you an idea of mine about prizes at school."

Doctor—"Well, madam, what is it?"

Mother—"I think the results would be beneficial in every way if prizes were offered: First, for the highest attainments in good manners—that is, in the daily and constant exposition of *true* politeness, which we all know cannot be practiced only when the person has a pure, good heart. Next, prizes for moral attainments, as regards the cultivation of an amiable temper, of unselfish, noble principles, and a disposition to do good deeds—in brief, to emulate a perfect moral character in all its attainments. Such ambition as this would be no tax upon the mental or physical faculties, and instead of doing them an injury would really be beneficial to health of body and mind, and if pupils tried to excel in moral worth, they would be so pure and conscientious, they would also try to excel in mental achievements without anticipation of any prize but the approval of a good conscience. What do you think of my idea?"

Doctor—"I think, madam, it is a very good one, and at the same time very unusual, and, as you put it, also contains both medical and moral philosophy, though some might object that moral ambition should have no prize in view, and be emulated only for its intrinsic worth. While your idea is a very good one, I do not know how it would be regarded in connection with our present systems of education."

Mother—"Neither do I, and only mention it for what it is worth. But, Doctor, I think there are other causes for the ill health of so many of our girls. Did you not, a few moments ago, mention clothing as one of them?"

Doctor—"Yes, madam, I am convinced that ignorance or obstinacy of parents on the subject of clothing is a fruitful source of ill health among girls as well as women. It is true our bodies receive warmth from the food we eat, but as clothing prevents heat, it should be used as an assistant to the food in giving protection to the body against changes of temperature of the weather, which is done upon the scientific principles of conduction and reduction of heat. All girls of the proper age should learn this at school, and the knowledge will enable them to do more than their mothers in selecting material as regards texture and color suitable for cold or warm weather."

Mother—"In warm weather I suppose we should have the material for clothing that will most readily reflect the sun's rays, and in the winter season that which is a bad or non-conductor."

Doctor—"Yes, madam, and girls will also then learn the various tortures, the loss of health, and damage to the beauty of form and features to which the vagaries of fashion subject the great majority of their sex. From early girlhood the waist is compressed with corsets that impede respiration and the action of the heart, and cause displacements of the organs of digestion and other functions of vital importance to beauty as well as health."

Mother—"As to these waspish-waists, I never could see any beauty or symmetry in them, leaving out the question of health and long life."

Doctor—"No, madam, there cannot be, according to the natural laws of grace and beauty. We all know that the lines of beauty and symmetry are in curves, not angles, and I am confident that corsets destroy the symmetry of a girl's waist and shoulders, as well as cause serious damage to her health. This can be readily seen by taking up a book upon physiology and comparing the out-lines of a body that has never been disfigured by compression with one that has."

Mother—"Yes, I have noticed this in those cuts, and in the former figure. I could see the sloping shoulders and graceful curves of the waist that are so essential to beauty of form in woman. If physicians and parents would impress it upon our girls that it is impossible for them to be beautiful without perfect health, I am sure they would be far more careful about avoiding every kind of illness, and would quit the reckless manner they have in disregarding so many things that would preserve their beauty and vigor."

Doctor—"I think they would, madam; it is natural for a woman to wish to be beautiful, even on to old age, if possible, and it is also right that she should have this desire, and should endeavor to cultivate her beauty in every proper manner. The only right way to do this is that designed by the Creator, and it is the only one that will promote and preserve real beauty of face and form. A woman who destroys her health by the silly mandates of fashion and society, cannot be the mother of healthy, bright and beautiful children, and she is also unfitted to rear them, and train them to become the truest and noblest types of men and women."

Mother—"Yes, I know that is true, and I have seen so many sad instances to confirm its truth."

Doctor—"Our ladies of fashion and those in easy circumstances do not take enough active, vigorous exercise, especially out of doors. Taking a short walk upon the streets or a daily drive is not sufficient to keep a woman in good health, nor to preserve her grace and beauty. At the majority of the girls' schools, if the pupils are allowed to engage in active exercise at all, the tyranny of dress and fashion seriously prevents the freedom of motion that is so essential to the good effects of the recreation. This is true, especially in calisthenics, that is now practiced in so many of these institutions. As Dr. Richardson, of London, says, the school-girl of the present day is taught to curb her joyous and natural spirits, to regard innocent recreation and any kind of romping as unlady-like, and to indulge in no exercise except that which is allowed by a prison-like routine, so that when she leaves school with a long list of showy accomplishments, all her physical energies have been sacrificed to a genteel deportment, and she is a ready victim to all kinds of nervous disorders."

Mother—"What kind of vigorous out-door exercise, Doctor, do you think is suitable for girls to have?"

Doctor—"There are several, madam, and among them, battle-door and shuttle-cock, lawn tennis, the game called rackets, and where the facilities are at hand, swimming, rowing and riding horseback are all excellent and vigorous recreation for girls of suitable age and growth, and also for ladies. These all conduce to expansion of the chest, an upright carriage and general strength of what is called the upper torso which will give full oxygenation of the blood, and thus keep the system in general good health."

Mother—"Do you think the skipping rope is a healthy exercise for girls?"

Doctor—"No, madam, not as it is invariably indulged in—on the contrary, it does much injury, and in some instances has destroyed health or caused sudden death. If girls would engage in the exercise in moderation and at the hours between breakfast and noon, it would perhaps do healthy girls no harm, but as I have said, they invariabl carry it to excess."

Mother—"Yes, I have often seen it done in my own school days; indeed, I have known girls to faint in the skipping rope, and be sick for weeks afterwards."

Doctor—"Very true, madam, I have heard of such instances. In all cases exercise of any kind, to be beneficial, should never be pursued to such an extent as to render it laborious, debilitating or exhaustive. This should be carefully considered; also the health of the person engaged in it. Delicate girls cannot take as much exercise as those in robust health, and neither can be truly benefitted by it, if the clothing is such as to interfere with the utmost freedom of motion of all parts of the body, and this cannot be had with high-heeled shoes and tight fitting garments."

Mother—"Parents and teachers will certainly learn, and before very long, how important it is to pay more attention to the physical recreation of our girls, instead of taxing their brains with a confusion of ideas that they soon forget, and also spending time and labor upon some showy accomplishments, for which the pupils often have no taste or capacity."

Doctor—"Yes, madam, I think you are correct in part, but as my time is out, I must bid you good morning."

